# The Influence of Strontium on Bone Tissue Metabolism and Its Application in Osteoporosis Treatment

**DOI:** 10.3390/ijms22126564

**Published:** 2021-06-18

**Authors:** Barbara Kołodziejska, Natalia Stępień, Joanna Kolmas

**Affiliations:** Department of Analytical Chemistry, Faculty of Pharmacy, Medical University of Warsaw, 02-097 Warsaw, Poland; barbara.kolodziejska@wum.edu.pl (B.K.); natalka.stepien-1999@wp.pl (N.S.)

**Keywords:** bone, osteoblasts, osteoclasts, strontium, osteoporosis, biomaterials, bone regeneration

## Abstract

Osteoporosis is a chronic disease characterized by low bone mass caused by increased bone turnover and impaired bone microarchitecture. In treatment, we use antiresorptive or anabolic drugs, which usually have a unidirectional effect, i.e., they inhibit the activity of osteoclasts or stimulate the effect of osteoblasts. Strontium ranelate is an anti-osteoporosis drug with a unique mechanism of action (used primarily in postmenopausal women). Unlike other medicines, it has a multidirectional effect on bone tissue, intensifying osteoblastogenesis while inhibiting osteoclastogenesis. It turns out that this effect is demonstrated by strontium ions, an element showing physical and chemical similarity to calcium, the basic element that builds the mineral fraction of bone. As a result, strontium acts through the calcium-sensing receptor (CaSR) receptor in bone tissue cells. In recent years, there has been a significant increase in interest in the introduction of strontium ions in place of calcium ions in ceramics used as bone replacement materials for the treatment of bone fractures and defects caused by osteoporosis. The aim of this study was to summarize current knowledge about the role of strontium in the treatment of osteoporosis, its effects (in various forms), and the ways in which it is administered.

## 1. Introduction

Proper bone remodeling is maintained due to a balance between two opposing mechanisms: bone formation and bone resorption. Osteoblasts, which are responsible for production of components of the bone intercellular substance, take part in the process of bone formation. Osteoclasts, in turn, are responsible for the bone resorption process. By secreting hydrolytic enzymes, osteoclasts dissolve bone components and phagocytose them, thereby destroying bone tissue [1,2]. The quantitative of resorptive processes over bone formation processes causes an increase in bone turnover, which results in a gradual reduction in bone mass, a decrease in mechanical strength, and a simultaneous increase in the risk of bone tissue fractures. The predominance of resorptive processes occurs primarily in the elderly population, especially in postmenopausal women, who also experience reduced oestrogen secretion. Pharmacological treatment of osteoporosis is usually associated with administration of antiresorptive drugs—mainly bisphosphonates—but also denosumab or calcitonin and anabolic drugs (parathyroid hormone analogues).

A unique drug that affects both the resorption and bone formation process is strontium, most often administered in the form of ranelate (SrRan). Strontium is an element from group II of the periodic table, discovered in the 19th century. In nature, Sr occurs as a mixture of four stable isotopic forms: _88_Sr (82.6%), _86_Sr (9.9%), _87_Sr (7.0%), and _84_Sr (0.6%). This metal is easily oxidized to form strontium oxide; therefore, Sr does not naturally exist in free form. Celestine (SrSO_4_) and strontianite (SrCO_3_) are the main minerals, which are present in nature. [3].

In the human body, strontium is a trace element. The daily strontium intake by an adult is approximately 4 mg. The main sources of this element are leafy vegetables, grains and dairy products (amounting to about 1.2–2.3 mg/day). Drinking water provides approximately 0.7–2 mg/day [3]. Strontium is poorly absorbed by the body, with an absorption level of approximately 25–30% [4]. Most of the absorbed element is found in the skeleton, but its content is only 0.035% of the calcium content [5]. In the organism, Sr is probably built into the crystals of biological apatite. It can form carbonate, citrate, and lactate salts, and it can also bind with calcium-transporting proteins.

The role of strontium in the human body is not fully understood and has yet to be fully explained [3]. This element, however, has a chemical similarity to calcium. For this reason, the properties and biological functions of these elements are also similar. Many animal studies have shown that strontium can replace calcium in some physiological processes, such as muscle contraction, blood clotting, and secretion of certain hormones. However, these processes are less intense than induction with calcium ions. Strontium can also be incorporated into bone cells, which increases their density and reduces the risk of developing osteopenia and osteoporosis. However, intravenous administration of a high dose of Sr^2+^ contributes to the occurrence of hypocalcaemia, caused by an increase in renal excretion of Ca^2+^ ions [6].

The aim of this study was to review current knowledge of the role of strontium in bone metabolism and to discuss the possibility of using its properties in the treatment of osteoporosis, with particular emphasis on the potential benefits and losses resulting from its use.

## 2. Mechanism of Strontium’s Influence on Bone Tissue

Due to the physical and chemical similarity of strontium and calcium, the processes of Sr interaction with bone tissue are based on mechanisms that take place physiologically with the participation of calcium. Both elements accumulate in plasma and extracellular fluids, soft tissues, and the skeleton. The rate at which strontium is incorporated into bone is very similar to that at which calcium is incorporated [4]. On the other hand, the results of studies in which strontium ranelate was administered to patients with postmenopausal osteoporosis indicate that the distribution of this element in bone tissue is heterogeneous. Higher strontium content is found in the trabecular bone, compared to the cortical bone. This may be due to a higher surface area-to-volume ratio or an increased rate of trabecular remodeling [7].

Strontium has a two-way effect on bone tissue. On the one hand, this element promotes formation of new bone tissue. By affecting osteoblasts, it increases their proliferation and differentiation. This is indicated by an increase in the expression of osteogenic genes such as *Runx*, *ALP*, *BSP*, and *BGP* [8]. Strontium enhances the synthesis of bone matrix proteins, which takes place in osteoblasts [9]. It also inhibits the apoptosis of these cells, which promotes osteoblast survival. On the other hand, strontium also affects osteoclasts. It inhibits the formation and differentiation of these cells and promotes their apoptosis, resulting in a reduction in bone resorption [8]. The mechanism by which strontium affects bone has yet to be fully explained. However, the similarity of strontium and calcium suggests that particular processes take place primarily through the calcium-detecting receptor CaSR [10].

### 2.1. A Calcium-Sensing Receptor

CaSR belongs to the family of G protein-coupled receptors. Its structure contains an extracellular (N-terminal) domain (which is a ligand-binding site), seven transmembrane domains, and an intracellular (C-terminal) domain [10]. The role of this receptor is to maintain proper calcium ion homeostasis in our body. CaSR is able to react to very small fluctuations in the concentration of calcium ions and activate a cascade of signals that facilitate restoration of the normal level of this ion [11]. These mechanisms are involved in the control of parathyroid hormone secretion by the parathyroid glands and the regulation of calcium ion reabsorption in renal tubules [12]. Many studies have shown that this receptor is also found in osteoblasts and osteoclasts, as a homologue of CaSR found in the parathyroid glands [8,13]. In bone tissue, CaSR regulates the concentration of calcium and the processes taking place in bone cells. Apart from calcium ions, other di- and trivalent cations (including the aforementioned Sr^2+^) can also activate CaSR [11].

### 2.2. The Influence of Sr^2+^ on Osteoblasts

Osteogenic cells—osteoblasts—are responsible for the process of osteogenesis. They are derived from marrow stromal mesenchymal stem cells (MSCs). They are also responsible for producing intracellular components of bone: collagen type I, proteoglycans, osteocalcin, osteonectin, osteopontin, and sialoproteins. The key transcription factors responsible for correct differentiation of osteoblasts are *Runx2* and *Sp7* (Runx2-dependent) [14]. Administration of strontium shows increased expression of key osteoblastogenesis genes by activating multiple signaling pathways. This promotes the differentiation and proliferation of pre-osteoblasts and osteoblasts, which results in an increase in the rate of bone formation and the synthesis of collagen and non-collagen proteins in the bone matrix [5]. At the same time, the mechanisms that inhibit osteoblast apoptosis increase their survival, which also has a positive effect on bone formation.

In addition to osteoblasts, MSCs have the ability to differentiate into other types of cells, including into adipocytes, chondrocytes, and myocytes. The transcription factor *Runx2* is responsible for differentiation of osteoblasts, while the expression of *PPAR-γ2* stimulates differentiation of MSCs into adipocytes. Strontium is a factor regulating the differentiation of MSCs. It causes a decrease in *PPAR-γ2* expression, thus inhibiting the adipogenesis process. At the same time, it increases expression of *Runx2*, favouring the differentiation of MSCs into osteoblasts. Strontium interacts through the canonical and non-canonical Wnt signaling pathways, as well as through the activation of *NFATc*. Administration of strontium in the form of ranelate increases expression of Wnt5a mRNA, which contributes to activation of the canonical Wnt signaling pathway. Consequently, this, in turn, increases expression of *Runx2* while inhibiting expression of *PPAR-γ2*. Wnt5a also activates a non-canonical signaling pathway, leading to activation of a cascade of subsequent signaling pathways, including *NFATc*. *NFATc* signaling regulates the transcription factor *Maf*, which controls expression of *Runx2* and *PPAR-γ2*. Activation of the *NFATc/Maf* pathway leads to a decrease in *PPAR-γ2* expression and *Runx2* activation, stimulating osteoblastogenesis. Strontium, through the Wnt and *NFATc/Maf* pathways, promotes the differentiation of MSCs into osteoblasts, which results in an increase in bone formation and an increase in bone mass [10,15].

Another mechanism that increases osteoblast differentiation is based on increased *COX-2* expression [8]. The cyclooxygenase enzyme is responsible for synthesis of prostaglandins. By increasing the production of *PGE2*, it stimulates bone formation. Studies involving mice in which *COX-2* expression was turned off showed decreased bone density, which confirms that this enzyme affects the functions of bone cells. *COX-2* also regulates expression of *Cfba-1* (belonging to the *Runx* family) and osterix, responsible for the differentiation of osteoblasts. In mice that did not express *COX-2*, there was a decrease in the expression of these genes, which resulted in decreased differentiation of osteogenic cells and abnormal bone repair [16]. Wnt signaling plays an important role in the regulation of osteoblast function. Wnts are glycoproteins that mediate intracellular signaling. They regulate many important cellular processes, which include morphogenesis, differentiation, proliferation, survival, apoptosis, polarization, and gene expression. Wnt binds to specific Frizzled receptors or receptors from the LRP family (included in the low-density lipoprotein). The receptor, in turn, binds to the Dishevelled protein in the case of the Frizzled receptor, or the axin in the case of the LRP receptor. As a result, the canonical pathway associated with activation of β-cathinone and its translocation from the cytoplasm to the nucleus is stimulated, where it binds to the *TCF/LEF* transcription factors, enabling intensification of the expression of genes promoting osteoblastogenesis [17,18].

Strontium via CaSH stimulates the *PI3K/Akt* signaling pathway. Akt activation occurs through the phosphorylation of this kinase by *PI3K*, which leads to the phosphorylation of β-cathinone and stimulates the canonical Wnt pathway [19]. As a result, the expression of pro-apoptotic genes and genes enhancing differentiation of osteogenic cells is increased. Regulation of the Wnt pathway can also be regulated by sclerostin. This is a protein produced by mature osteocytes, which reduces the differentiation and proliferation of osteoblasts, leading to inhibition of bone formation [20]. Thus, sclerostin is an inhibitor of the canonical Wnt pathway. It binds to the Frizzled/LRP receptor, preventing Wnt from attaching to the receptor. Strontium reduces expression of sclerostin, which prevents inhibition of the Wnt pathway and stimulates the bone formation process. These mechanisms indicate that strontium activates the canonical Wnt pathway by stimulating the *PI3K/Akt* pathway and reducing sclerostin expression. Thus, it promotes the differentiation of osteoblasts and, by inhibiting apoptotic processes, supports their survival [21].

Strontium also stimulates the *Cn-NFATc* signaling pathway in osteoblasts [8]. This element activates calcineurin (a protein with phosphatase activity), which acts on *NFATc* by dephosphorylation. In an inactive state, this transcription factor is found in the cytoplasm, but after activation by calcineurin, it translocates to the nucleus [22], where it attaches to the *Runx* and *ALP* genes, which promotes osteoblastogenesis and increases their expression [8]. Activation of the *Cn/NFATc* pathway may also increase expression of Wnt proteins, which leads to an increase in the concentration of β-cathinone in cells and an increase in expression of genes controlling the differentiation and proliferation of osteoblasts [23].

The genes that regulate osteoblast function are also regulated via the *Ras/MAPK* pathway. Ras is a protein with GTPase activity. It binds to the Raf kinase, causing it to activate. This induces the phosphorylation of subsequent MEK1/2 kinases, which leads to activation of the effector kinases ERK 1/2 and p38 [24]. Strontium enhances the *Ras/MAPK* signal, which leads to phosphorylation and enhances the transcriptional activity of *Runx2* in pre-osteoblasts. This results in an increase in osteoblast differentiation and an increase in the expression of osteogenesis markers (*ALP*, *BSP*, and *BGP*), which reflects increased osteoblastic activity and increased mineralization. This indicates that stimulation of the *Ras/MAPK* pathway positively influences differentiation of osteogenic MSCs into osteoblasts [25].

Unlike calcium ions, which trigger an immediate signal cascade upon binding to CaSR, strontium ions act with a delay, activating signaling pathways one hour after binding to the receptor, which appears to be critical to cell function [8].

### 2.3. The Influence of Sr^2+^ on Osteoclasts

Osteoclasts are derived from hematopoietic stem cells. They differentiate into immature pre-osteoclasts, and then several precursor cells fuse to form mature polynuclear osteoclasts [26,27]. These cells are responsible for bone resorption. In osteoporosis, bone resorption processes exceed those of formation, indicating excessive osteoclast activity. Strontium inhibits bone resorption processes, affecting the activity of osteoclast cells [4] by reducing their differentiation and intensifying the apoptosis process of mature osteoclasts [8].

The differentiation and function of osteoclasts is under the control of osteoblasts. They secrete factors that regulate the maturation of bone-resorbing cells. These factors include M-CSF and RANKL [23]. M-CSF enhances the proliferation of osteoclast precursors and also inhibits their apoptosis. Successive stages of osteoclast maturation are regulated by the *RANK/RANKL/OPG* pathway. RANKL is a protein produced by osteoblast precursors, osteoblasts, and osteocytes. At the same time, RANKL is a ligand for the RANK receptor, which is found on the surface of osteoclasts and their precursors [28]. Combining RANKL with RANK triggers a signal cascade, activating the *NF-κB*, *MAPK* and *PI3K/Akt* pathways, stimulating osteoclastogenesis [29]. Osteoprotegerin (OPG) is a receptor from the TNF superfamily and is synthesized by osteoblasts. OPG represents the “decoy receptor” for RANKL and reduces its binding to RANK [28]. Strontium, acting through the CaSR receptor on osteoblasts, increases production of osteoprotegerin and reduces expression of RANKL mRNA, which prevents the binding of RANKL to RANK [10], leading to the inhibition of osteoclastogenesis [30].

**Figure 1:**
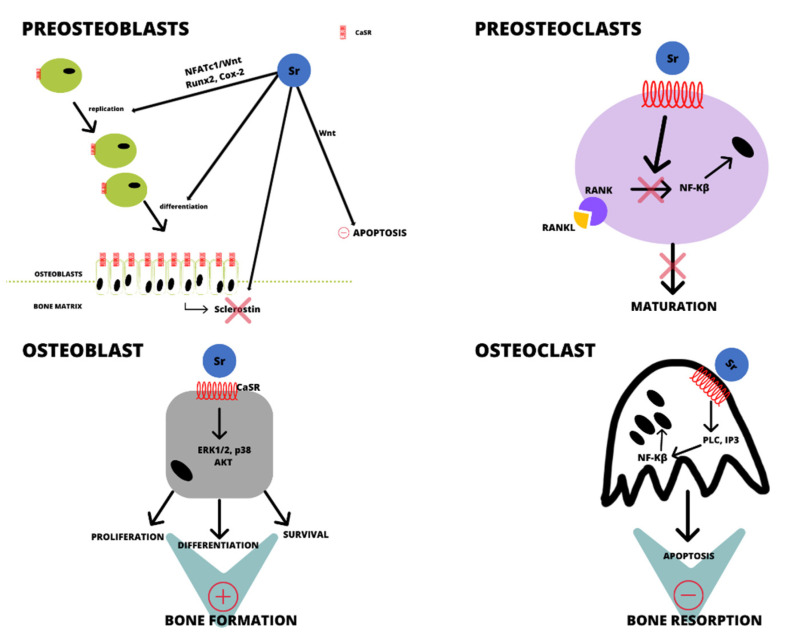
Effect of strontium on the function of osteoblasts and osteoclasts. Plus signs stimulation and minus signs inhibition.

Strontium also reduces bone resorption by enhancing the apoptosis of mature osteoclasts. Strontium mediated by CaSR changes the conformation of G proteins associated with the receptor and stimulates their activity. Activation of *Gαq* leads to production of PLC, which converts PIP2 to DAG and IP3, increasing the concentration of IP3 in cells [8]. This leads to activation of NF-κB through translocation of this factor to the nucleus, resulting in the stimulation of apoptosis in mature osteoclasts. The interaction of strontium ions with CaSR leads to activation of many types of G proteins, resulting in multiple pathways of osteoclast apoptosis. However, the main regulators of the apoptosis process of mature osteoclasts are PLC, PKCII, and NF-κB [31]. The overall effect of strontium on the function of osteoblasts and osteoclasts is shown in Figure 1.

## 3. Strontium in the Treatment of Osteoporosis

### 3.1. Strontium Ranelate in Osteoporosis

**Figure 2:**
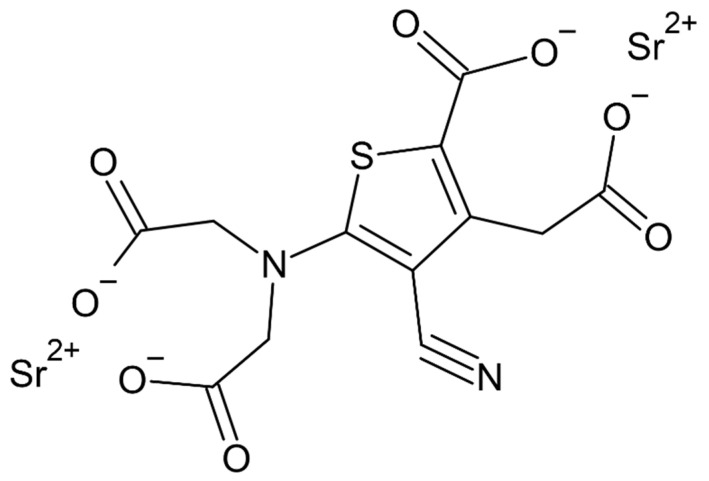
Strontium ranelate.

From the beginning of the 21st century, strontium ranelate (SrRan) began to play an important role in the treatment of osteoporosis. It is a dual-action agent, which acts on bone cells and has been used and registered in over 70 countries since 2004 [32,33]. On the basis of many studies, the effectiveness of this drug substance has been shown, mainly in the treatment of postmenopausal osteoporosis in women, as one of the first-choice drugs [34]. Chemically, strontium ranelate is a salt of thiopheneacetyl acid, where two stable divalent strontium atoms are linked to ranelic acid (Figure 2) [9,19]. Strontium in the form of such a salt was first introduced in 1993 by Marie et al. [35].

Contrary to other drugs used, which act by stimulating the activity of osteoblasts or suppressing the activity of osteoclasts, SrRan has a multidirectional effect on bone tissue. SrRan affects the cells of bone tissue, which significantly affects remodeling of the mineral fraction of bone tissue. This results in an increase in the volume of cortical and trabecular bone, stimulates bone formation, and improves its microarchitecture. Due to changes in the properties of the bone matrix and bone mineral density, SrRan prevents bone loss, thereby increasing its mechanical strength and improving the overall quality of bone tissue [19,36]. SrRan stimulates the activity of osteoblasts while reducing differentiation and the resorptive effects of osteoclasts and increases the synthesis of collagen and non-collagen proteins [9].

Many studies have also shown that ranelate treatment is effective in preventing both vertebral and non-vertebral fractures (e.g., hip fractures). The SOTI (Spinal Osteoporosis Therapeutic Intervention) study found a reduction in the incidence of spine fractures in postmenopausal women after just one year of treatment. This decrease, compared to the placebo control group, amounted to 49%. A three-year double-blind study showed a 41% reduction in the incidence of new spine fractures, compared with a placebo group. TROPOS (treatment of peripheral osteoporosis) studies have shown the effective anti-fracture effect of ranelate on non-vertebral fractures within the first three years of use. These fractures were reduced by 16% [37,38,39].

The recommended dose for treatment of osteoporosis is 2 g of strontium ranelate daily, taken as an oral suspension [40]. In clinical trials, the above dose was used for three years, and in follow-up studies, long-term results were noted after treatment lasting 5 to 10 years [41]. Strontium ranelate was, at one point, withdrawn from treatment due to reports of serious side effects. Information has emerged about the growth of cardiovascular risk and non-fatal myocardial infarctions. Recently, the EMA (European Medicines Agency) has issued an overview of how strontium ranelate can be used with many restrictions on its use [38,42]. For this reason, an alternative route for administration of strontium ranelate has been sought in order to minimize the serious side effects associated with oral administration. Consequently, Sr-enriched biomaterials have been looked at in particular. Interestingly, it has also been proven that strontium ranelate has a positive effect on improving implant fixation [43], which may also be useful in the design of this type of biomaterial.

**Figure 3:**
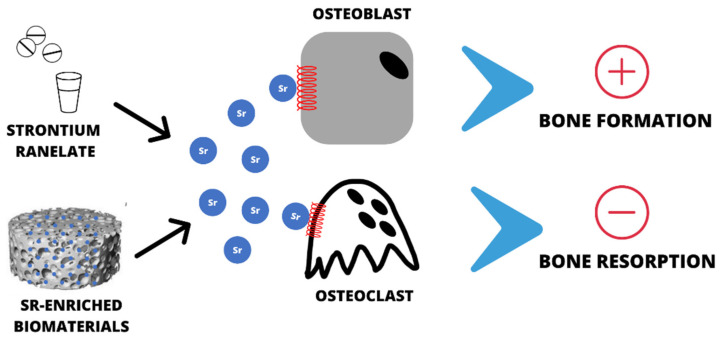
Different routes for administration of strontium ions.

Attempts have been made to administer strontium ranelate locally in the form of an injection, which has also brought about beneficial results [44]. Other attempts have been made to introduce the drug substance SrRan together with titanium implants [45], but the results were found to be inconclusive. SrRan has also been combined with polymer materials—poly(lactic acid)—and their effectiveness in vitro has been tested with good results [46]. Certainly, attempts to administer strontium ranelate locally are beneficial and worth considering (Figure 3).

### 3.2. Local Administration of Strontium Ions

In recent years, widely understood ceramic biomaterials have played an important role in the treatment of bone diseases. Many studies, both in vitro and in vivo, have confirmed high bioactivity and a large share in the formation of new bone tissue by such materials as calcium silicate, calcium phosphate (hydroxyapatite), bioglasses, bone cements, or polymer-based implants. Considering the highly beneficial effects of medicines containing strontium ions, as well as biocomposites for bone regeneration materials, attempts have been made to combine these two substances. Strontium-enriched biomaterials are an extremely advantageous solution, which would allow local delivery of strontium ions while minimizing the side effects that occur with systemic administration. Moreover, there are numerous reports that the bioceramic material itself also gains new, valuable physicochemical properties due to the presence of strontium ions in the apatite structure [47,48,49,50].

**Figure 4:**
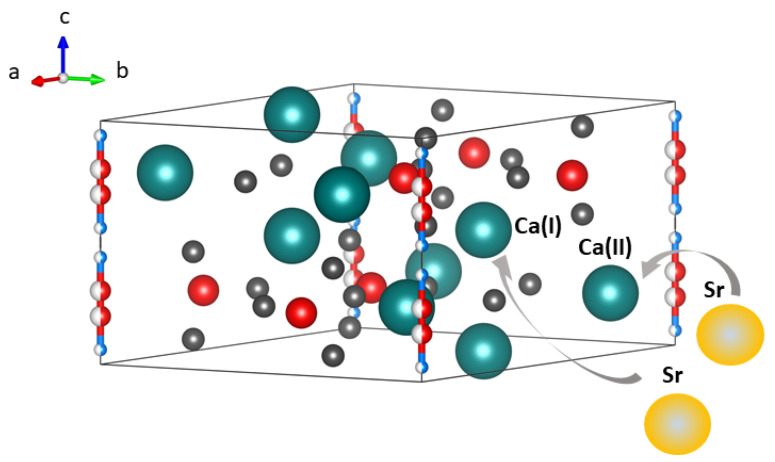
Incorporation of strontium ions into the structure of calcium hydroxyapatite (a-, b- and c-axes orientation).

Calcium phosphate-based ceramic biomaterials, due to their great similarity with natural bone tissue, are intensively studied to combine them with strontium ions. Strontium ions can be incorporated into the structure of calcium phosphate apatites in two ways (Figure 4). The first (faster) is adsorption on the surface of the mineral. The second takes advantage of the chemical similarity between Sr and Ca. Sr ions easily incorporate into the crystalline lattice structure, replacing calcium. Incorporation of strontium ions into the apatite structure is a slightly slower process and involves the formation of bonds between phosphate groups and strontium ions [51,52,53,54,55,56,57]. The first reports on this topic appeared as early as 1870, when the incorporation of Sr into bone structure was noted in animals fed with strontium phosphates and strontium carbonates [58,59]. The amount of Sr incorporated into the structure depends on many factors, e.g., on the age of the bone tissue or the type of bone (spongy or compact) [56,60,61]. Research shows the presence of Sr in newly formed biological apatite in an amount from 5 to 10% of Ca replaced by Sr in the lattice [61,62].

Orally administered strontium ranelate does not significantly affect bone formation in vivo because it does not reach a sufficient concentration near this tissue, assuming a standard drug supply and a normal calcium-rich diet. Local release of strontium near the bone defect can provide osteoblasts with the appropriate concentration of this element, leading to enhanced bone formation and osseointegration [8]. Besides the effect on bone cells, treatment with strontium ranelate has an effect directly on the mineralized bone matrix [41]. Research proves that biomaterials enriched with strontium ions differ physically and chemically when compared to biomaterials without Sr ions, e.g., in solubility and ion release kinetics. Some studies suggest that local Sr release is sufficient to stimulate bone formation without the presence of other osteogenic ions.

Due to the great potential of strontium in the treatment of osteoporosis and other bone diseases, ion-modified porous scaffolds containing strontium have become of great interest in recent years. Porous ceramics can also be treated as a DDS (drug delivery system) for strontium ranelate.

So far, several in vitro studies on animal models have been developed to test the effectiveness of strontium-enriched hydroxyapatite bioceramics [63,64,65,66]. Research has confirmed the therapeutic potential of such materials, indicating their effectiveness in the regeneration of bone tissue, high biocompatibility, and improvement of osteogenic abilities. In studies, strontium was introduced into the biomaterial so that it constituted 10% of the Ca content [63,65].

The kinetics of release of Sr ions from the apatite structure has also been investigated. In one study, where the subject of interest was bioglasses with embedded strontium ions, the kinetics increased linearly with increasing concentrations of Sr ions [67]. Another study found that doping with Sr accelerates the rate of apatite degradation and that this ion could be released when the matrix dissolves, allowing it to fulfil its therapeutic function [68].

To date, few studies have been carried out with human subjects, but the research at our disposal confirms the high biocompatibility of these types of materials. Korovessis et al. conducted studies using Sr-HA materials instead of polymethacrylate (PMMA) (routinely used in vertebroplasty) and proved the effectiveness of strontium ion material in post-traumatic spine fractures, although more patients and longer studies are needed. Sr-HA was resorbed and replaced with vertebral bone, and no new fractures were observed. Cheung et al. and Izci et al. also obtained favorable in vivo results [69,70,71].

Chen et al. described the effectiveness of taking SrRan orally with the implantation of materials modified with Sr ions [72]. The study proved that such a combination has a great influence on the improvement of osseointegration ability and positively influences the angiogenesis process.

Strontium ions have recently been integrated into bone cement. Rohnke et al. studied the kinetics of the Sr^2+^ release from bone cement [73]. Time of flight secondary ion mass spectrometry (ToF-SIMS) was used to determine the strontium diffusion coefficient in healthy and osteoporotic trabecular rat bone. Sr^2+^ dissipation is more advanced and therefore faster in osteoporotic bone, which could be due to different bone nanostructure. Using ToF-SIMS, it is proven that strontium is localized in newly formed bone regions and within the pre-existing osteoporotic bone [74,75,76].

Other biomaterials enriched with strontium ions are calcium silicate-based biomaterials [77]. Calcium silicate bioceramics have osteogenic activity and stimulate angiogenesis. Xing et al. developed Si-Sr/alginate hydrogels, which release Si and Sr ions in the same optimal concentrations range for osteogenesis activation. It stimulates not only the osteogenic differentiation of osteoblasts, but also the blood vessel formation in vivo [78]. Many other studies have confirmed the synergistic effect of Sr and Si, which has resulted in a beneficial effect on the bone tissue remodeling process [79,80,81,82].

Bioactive glasses are other interesting materials that could be used as bone substitutes. They are characterized by high bioactivity and biodegradability. Moreover, they accelerate bone formation. The addition of strontium to bioglasses improves the bioactivity and increases the quantity of the newly regenerated bone compared to the corresponding Sr-free biomaterials. In addition, bioglasses enriched with strontium ions accelerate the healing process [83,84,85].

Some composite scaffolds containing Sr ions have also been successfully used in bone tissue engineering. Quade et al. developed collagen/hydroxyapatite nanocomposites, where calcium was substituted with strontium. Strontium ions were released in a sustained manner from the modified scaffolds and the released Sr^2+^ doses have shown osteo-anabolic effects [86]. In turn collagen combinations with strontium-containing mesoporous bioactive glasses revealed bioactive properties as a result of the fast and conspicuous HA crystal deposition. The results of the histopathological tests showed high levels of bone formation in the Sr-bioglasses scaffolds [87,88,89]. Other polymer-based biomaterials are also used, e.g., strontium-graphene oxide in the collagen scaffold, polycaprolactone (PCL), or silk fibroin [90,91,92,93].

Attempts to modify biomaterials with strontium ions are still valid. Recent papers report the use of materials such as iron oxide nanospheres-hydroxyapatite-strontium@collagen (IONSs-HA-SR@C) [94] or macroporous iron foams coated with strontium [95]. Such materials appear to be particularly useful in the osteoporotic fracture defect healing, as research shows significantly increased bone formation at the implant interface with increased osteoblast and decreased osteoclast activity. In addition, the IONSs-HA-SR@C showed anti-bacterial activity and a significant efficacy in infection treatment. Last but not least, these materials have excellent biocompatibility.

## 4. Conclusions

The mechanism of action of strontium is still not fully understood, which is a significant problem in modern science. However, the research that has been conducted to date has brought us closer to understanding the mechanism by which strontium ions work in the bone formation process. It seems that administering Sr locally can be very effective, with fewer side effects than systemic administration, and it can significantly improve the osseointegration of bone implants.
